# Long non‐coding RNA H19 promotes TDRG1 expression and cisplatin resistance by sequestering miRNA‐106b‐5p in seminoma

**DOI:** 10.1002/cam4.1871

**Published:** 2018-11-14

**Authors:** Jingchao Wei, Yu Gan, Dongyi Peng, Xianzhen Jiang, Riko Kitazawa, Yali Xiang, Yingbo Dai, Yuxin Tang, Jianfu Yang

**Affiliations:** ^1^ Department of Urology The Third Xiangya Hospital of Central South University Changsha China; ^2^ Department of Urology Xiangya Hospital of Central South University Changsha China; ^3^ Department of Diagnostic Pathology Ehime University Hospital Toon Japan; ^4^ Department of Health Management Center The Third Xiangya Hospital of Central South University Changsha China; ^5^ Department of Urology The Fifth Affiliated Hospital of Sun Yat‐sen University Zhuhai China

**Keywords:** chemotherapy, drug resistance, long non‐coding RNA, microRNA, seminoma

## Abstract

The role of TDRG1 in tumorigenesis and the progression of seminoma, as well as its role in regulating chemosensitivity of seminoma to cisplatin through the PI3K/Akt/mTOR signaling pathway, has been previously defined. However, the detailed mechanism underlying TDRG1 expression and concomitant chemoresistance conditions are unknown. Furthermore, it has been reported that non‐protein‐coding RNAs play an important role in a variety of vital processes including cellular chemosensitivity. However, the role of non‐protein‐coding RNAs in regulating the chemosensitivity of seminoma remains unknown. In this study, using microarray analysis, we found that long non‐coding RNA H19 was upregulated while miRNA‐106b‐5p was downregulated in an established cisplatin‐resistant TCam‐2 cell line. Moreover, H19 acts as a miRNA‐106b‐5p sponge and thus impairs the function of miRNA‐106b‐5p on its target gene, TDRG1. Based on these findings, we propose that H19 promotes the expression of TDRG1 by sequestering miRNA‐106b‐5p and uses this mechanism to facilitate cell survival in cisplatin‐based chemotherapeutic conditions. These findings elucidate the mechanisms, at least partially, applied to deregulate TDRG1 and cisplatin sensitivity, and may provide new therapeutic possibilities for chemoresistant seminoma.

## 1 INTRODUCTION

Despite accounting for only 2% of all malignancies in males, testicular tumors pose a serious threat to young men, as the incidence of testicular tumors has been increasing worldwide for decades.^1^ About 95% of these malignancies are testicular germ cell tumors, which arise from germ cells and can be classified as seminoma and non‐seminoma.^2^ Fortunately, seminomas have a high cure rate, especially after the introduction of cisplatin (cis‐diamminedichloroplatinum II, CDDP) as a therapeutic treatment in the mid‐1970s.^3^ Though CDDP is a highly effective therapy for most seminoma patients, late relapses of testicular cancers (>2 years interval between the initial treatment and recurrence) with chemoresistant features have been reported.^4^ By improving the overall chemosensitivity of seminoma, it is more likely that patients who develop testicular cancer with seminoma chemoresistance will survive. Furthermore, a higher sensitivity to CDDP may reduce the dose of drug required, as well as decrease the chance of developing of long‐term complications.

Previously, we have found that the sensitivity of seminoma to CDDP is regulated by TDRG1 (testis developmental related gene 1), an oncogene that is exclusively expressed in testis and can promote the proliferation and progression of human seminoma cells through PI3K/Akt/mTOR signaling.^5^, ^6^ However, the mechanisms underlying the gain of function of TDRG1 in seminoma and CDDP‐resistance context remains to be determined. We believe that elucidating the mechanisms enhancing TDRG1 expression in seminoma may help us further understand the progression of this disease and how tumor cells survive under CDDP treatment.

Solid evidences have uncovered the crucial role of non‐protein‐coding RNAs (ncRNAs) in gene regulation. This is further supported by results from RNA‐seq data, in which a large proportion of the genome has been found to be transcribed into ncRNAs under various conditions.^7^ Among ncRNAs, both microRNAs (miRNAs) and long non‐coding RNAs (lncRNAs) have emerged as essential and necessary molecules in tumor biology and may participate in regulating drug resistance.^8^ miRNAs are short, non‐coding RNA molecules that regulate gene expression at a post‐transcriptional level. lncRNAs are non‐coding genomic transcripts longer than 200 nucleotides, whereby only a relatively small proportion have been reported to be functionally annotated.

In this study, we compared the transcriptome of a newly established CDDP‐resistant seminoma TCam‐2 cells and their parental origins so as to define two differentially expressed ncRNAs—miRNA‐106b‐5p and lncRNA H19. Not only did we report that the ncRNAs expression was confirmed, we also found that their interactions regulate the expression of TDRG1 and CDDP sensitivity in seminoma.

## 2 METHODS

### 2.1 Ethics statement

The Institutional Research Ethics Committee of The Third Xiangya Hospital of Central South University (CSU), Changsha, China, approved the study. All patients provided written informed consent, and all experiments adhered to the principles set forth in the Declaration of Helsinki.

### 2.2 Human tissue specimens and cell culture

Ten tumor specimens from patients with seminoma and 10 normal testicular tissues from donors were obtained from the Third Xiangya Hospital of CSU from June 2009 to June 2015. The human TCam‐2 cell line was kindly gifted by Dr Riko Kitazawa, Department of Diagnostic Pathology, Ehime University Hospital, Matsuyama, Japan. TCam‐2 cells were maintained in complete medium (Roswell Park Memorial Institute Medium‐1640 with 10% fetal calf serum) with 5% CO_2_ at 37°C. The TCam‐2/CDDP cell line was established from the TCam‐2 cell line by long‐term intermittent administration of low‐dose CDDP, as described by Kobayashi et al^9^

### 2.3 Quantitative real‐time PCR, immunoblotting, and immunohistochemistry

The procedures for these assays can be found as we have previously reported.^6^ For quantitative real‐time PCR (qPCR), total RNA was extracted using the miRNeasy Mini Kit (Qiagen, Valencia, CA) according to the manufacturer's instructions. For miRNA‐106b‐5p, small RNA U6 was used as an internal control, and the relative level of miRNA‐106b‐5p was normalized to U6 expresion.^10^ Relative H19 expression was normalized to housekeeping gene GAPDH. The relative levels of RNAs were calculated using the 2^−ΔΔCt^ method.^11^ For the immunoblotting assay, GAPDH protein was used as a loading control. The relative protein content was normalized to GAPDH. In immunohistochemistry (IHC) assay, the information for the primary antibodies is as follows: TDRG1‐antibody, 1:1000 dilution, Novus Biologicals, Littleton, CO; Ki‐67‐antibody, 1:100 dilution, GeneTex, Irvine, CA.

### 2.4 Cell viability assay and cell invasion assay

Cell viability assays and cell invasion assays were performed according to protocols as previously described.^5^ Briefly, before the absorbance was measured by the MTT assay at 590 nm, cells were treated with CDDP for 0, 12, 24, 48, and 72 hours, respectively. In cell invasion assays, cells were seeded on Matrigel‐coated polycarbonate in a transwell apparatus and the proportion of cells migrating from the upper compartment to the lower compartment was analyzed under the microscope after being incubated in the transwell plate at 37°C and 5% CO_2_ for 2.5 hours.

### 2.5 Cell cycle and cell apoptosis analysis

Cell cycle and cell apoptosis assays have been previously described.^6^ Briefly, cells were treated with CDDP for 72 hours and then stained with FL2 for cell cycle analysis or double stained with Annexin V and proliferation index (PI) for cell apoptosis analysis. Cell PI was calculated according to the distribution of cell cycle, as previously reported.^5^ Cells with Annexin V staining were considered to be undergoing apoptosis.

### 2.6 ShRNA‐H19 plasmids and miRNA‐106b‐5p mimics transfection

ShRNA‐H19 plasmids were purchased from Nkbiotechnology company (Changsha, China), which were constructed based on the pGPU6/GFP/Neo vector. shRNA‐H19 plasmids or negative control plasmids were transfected into tumor cells using Lipofectamine 3000 (Invitrogen, Carlsbad, CA) following the provided instruction. Synthetic miRNA‐106b‐5p mimics (Nkbiotechnology) or negative controls were also transfected using Lipofectamine 3000 (Invitrogen). Sequences of shRNA‐H19 inserts and miRNA‐106b‐5p mimics are listed in Table S1.

### 2.7 Mutation of TDRG1‐3′UTR and H19

The vectors with mutant 3′UTR (3′‐untranslated regions)‐TDRG1 or mutant H19 element were constructed by Nkbiotechnology company, using a QuikChange II Site‐Directed Mutagenesis Kit. Sequencing and qPCR were employed for validation. The detail information is provided in Data S1 and S2.

### 2.8 Luciferase reporter transfection and dual luciferase assay

Reporters of H19‐WT (wild type), H19‐MT, 3′UTR‐TDRG1‐WT, and 3′UTR‐TDRG1‐MT were constructed based on pmirGLO vector backbone. Briefly, TCam‐2 cells were seeded onto 48‐well plates at 5 × 10^4^ cells per well overnight. Reporter plasmids and Renilla plasmids were then co‐transfected with miRNA mimics or scrambled miRNA into TCam‐2 cells by using Lipofectamine 3000, according to the manufacturer's protocol (Invitrogen). After 48 hours, the cells were lysed using the Dual‐Glo Luciferase Assay Kit (Invitrogen) following the provided instruction before the dual luciferase activities were measured. Relative light unit of firefly luciferase activity was normalized to that of Renilla.

### 2.9 RNA‐binding protein immunoprecipitation assay

RNA‐binding protein immunoprecipitation (RIP) assay was performed using the EZ‐Magna RIP™ RNA‐Binding Protein Immunoprecipitation Kit (Millipore, Billerica, MA) according to the manufacturer's instructions. Briefly, TCam‐2 cell lysis was collected and then anti‐Ago2 antibody (Cell Signaling Technology, Beverly, MA) or anti‐IgG antibody (negative control; Sigma, St. Louis, MO) was used to prepare magnetic beads, which was followed by RNA purification. Immunoprecipitated H19 was analyzed by qPCR.

### 2.10 RNA pull‐down assay

RNA pull‐down was performed using the Pierce™ Magnetic RNA‐Protein Pull‐Down Kit (Thermo Scientific, Waltham, MA) according to the manufacturer's instructions. Briefly, scrambled miRNAs, miRNA‐106b‐5p‐WT, and miRNA‐106b‐5p‐MT were biotin‐labeled and transfected into TCam‐2 cells. After 48 hours, cells were collected to obtain cell lysis. Then, labeled RNA was captured using streptavidin magnetic beads. Binding of RNA‐binding proteins to RNA was carried out using RBP Enrichment Module. RNA‐protein compounds were subsequently eluted off and RNA was purified by TRIzol kit (Invitrogen), followed by H19 detection using qPCR.

### 2.11 Xenografts

The Department of Laboratory Animals, CSU, provided a total of 24 male BALB/c nude mice. The in vivo experiments were conducted in compliance with the recommendations in the Guide for the Care and Use of Laboratory Animals of CSU. Mice were subcutaneously injected with tumor cells (1 × 10^6^/mouse, 0.2 mL for each injection site) near the limbs. TCam‐2/CDDP cells with stable knockdown of H19 or TDRG1 were injected into the mice. To increase miRNA‐106b‐5p expression, tumors derived from TDRG1/CDDP cells were injected with miRNA‐106b‐5p mimics every 3 days for seven doses. For CDDP treatment, mice were intraperitoneally injected with CDDP (6 mg/kg body weight) every week for three doses after tumor cell inoculation. Mice were sacrificed 25 days after tumor cell inoculation and tumor lumps were excised for subsequent experiments, including immunoblotting (frozen in liquid nitrogen), H&E, IHC (fixed in 10% formalin), and qPCR (total RNA was isolated from fresh tissues).

### 2.12 Gene Expression Omnibus datasets studies

We analyzed the Gene Expression Omnibus (GEO) datasets using the GEO2R method^12^ (available at https://www.ncbi.nlm.nih.gov/geo/geo2r/). GEO2R performed comparisons on originally submitted data (processed and normalized) using the GEO query and limma R package.^13^

### 2.13 Statistical analysis

Data were presented as mean ± standard deviation of three or more independent experiments and analyzed using GraphPad Prism 6 (GraphPad Software, San Diego, CA). *T* test and analysis of variance were used to compare group means. Bivariate correlations were assessed using Pearson's correlation. *P* < 0.05 was considered to be statistically significant.

## 3 RESULTS

### 3.1 H19 and miRNA‐106b‐5p are tightly associated with TDRG1 in seminoma and CDDP‐resistant cellular context

**Figure 1:**
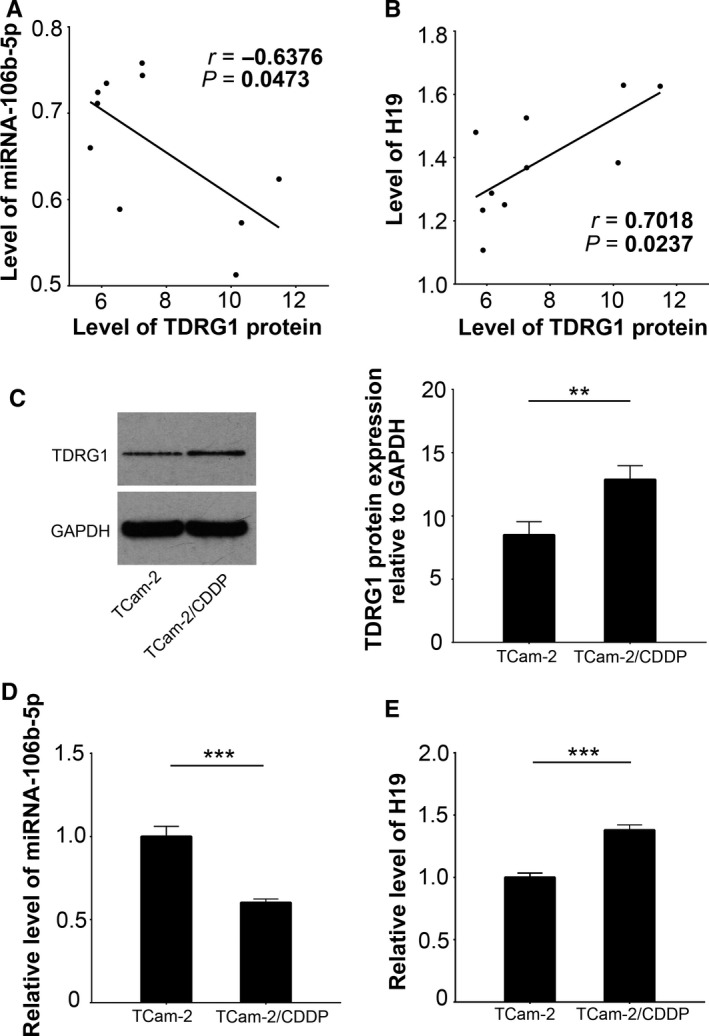
Both H19 and miRNA‐106b‐5p are associated with TDRG1 in seminoma and CDDP‐resistant cellular context. The expression of TDRG1 protein was measured by immunoblotting, while the expression of miRNA‐106b‐5p and H19 was tested by qPCR. The association of TDRG1 with miRNA‐106b‐5p (A) or H19 (B) in seminoma tumor tissues (n = 10) was then analyzed. TCam‐2/CDDP cells were derived from the parental cell origins, TCam‐2 cells, by chronic CDDP exposure. The expression of TDRG1 protein (C), miRNA‐106b‐5p (D) and H19 (E) was measured. H19, lncRNA H19; TDRG1, testis developmental related gene 1; qPCR, quantitative real‐time PCR; CDDP, cisplatin; TCam‐2/CDDP, CDDP‐resistant TCam‐2; **P < 0.01, ***P < 0.001

To uncover the expression profiles of miRNAs and lncRNAs in CDDP‐resistant seminoma, we successfully established a TCam‐2/CDDP cell line. Compared to the parental cells, TCam‐2/CDDP cells were more resistant to CDDP (Figure S1A,B). A comparative analysis of the expression profiles of lncRNAs and miRNAs between TCam‐2/CDDP cells and their parental origins was performed by microarray. The results indicated that, in addition to an increased ability to survive CDDP treatments, there was a significant change to the lncRNA and miRNA profile of the seminoma cells (Figure S2A,B). Among the differentially expressed ncRNAs, we decided to focus our interest on H19 (increased, logFC > 2, *P < *0.01) and miRNA‐106b‐5p (decreased, logFC < −2, *P < *0.01) based on the following reasons: (a) both ncRNAs have been previously reported to be associated with CDDP resistance^14^, ^15^, ^16^; (b) using two online tools (microrna.org and mirDIP), we found that miRNA‐106b‐5p was predicted to bind with TDRG1 mRNA, while H19 was predicted to bind with miRNA‐106b‐5p, according to StarBase and DIANA tools/LncBase Predicted; (c) bioinformatic analyses from the datasets in GEO implied a similar trend of expression changes of the two ncRNAs as found in our data (Figure S3A,B). To detect H19 and miRNA‐106b‐5p expression in seminoma, we collected 10 specimens from seminoma patients and 10 specimens of normal testicular tissues. When compared to the normal tissues, higher levels of TDRG1 protein (*P < *0.001) and H19 (*P < *0.01) and lower levels of miRNA‐106b‐5p (*P < *0.001) were detected in seminoma (Figure S4A‐D). As expected, the level of miRNA‐106b‐5p was negatively associated with TDRG1 expression in seminoma (Figure 1A, *P < *0.05), while H19 showed a positive correlation with TDRG1 expression (Figure 1B, *P < *0.05). The levels of H19 and miR‐106b‐5p in these samples presented a moderate trend of negative correlation (*r* = −0.3072, *P* > 0.05). As TDRG1 can regulate sensitivity of CDDP in seminoma cells,^6^ we hypothesized that both H19 and miRNA‐106b‐5p may play important roles in CDDP resistance. This is partially supported by the change of their expressions in the transition of TCam‐2 to TCam‐2/CDDP cells, during which the expression of TDRG1 (*P < *0.01) and H19 (*P < *0.001) was increased and the level of miRNA‐106b‐5p (*P < *0.001) was decreased (Figure 1C‐E).

### 3.2 H19 functions as an endogenous sponge of miRNA‐106b‐5p

**Figure 2:**
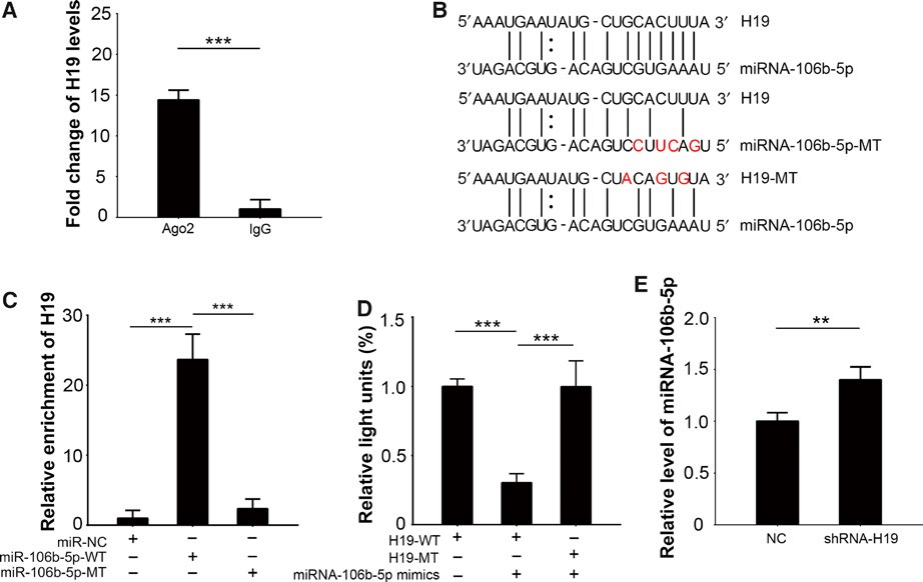
H19 functions as a miRNA‐106b‐5p “sponge.” A, RIP assays were performed using Ago2 antibody or IgG antibody (negative control), respectively. Eluted RNA fragments were used as templates to perform qPCR to measure the binding of H19 with these proteins. B, A schematic diagram of the predicted binding region of H19 and miRNA‐106b‐5p. The artificially mutated sequences in H19 and miRNA‐106b‐5p are also presented. C, RNA pull‐down assays were performed by transfecting with biotin‐labeled scrambled miRNAs, miRNA‐106b‐5p‐WT, or miRNA‐106b‐5p‐MT, respectively. Eluted RNA fragments were used as templates to perform qPCR to measure H19 enrichment. D, The luciferase reporters containing the H19 WT or the MT‐binding sequence were co‐transfected with miRNA‐106b‐5p mimics and Renilla plasmids into 293T cells, and then the luciferase activities were measured. Firefly luciferase activity was normalized to Renilla. E, H19 was silenced in TCam‐2 cells. The expression of miRNA‐106b‐5p was determined by qPCR. Ago2, argonaute‐2; IgG, immunoglobulin G; MT, mutant‐type; RIP, RNA‐binding protein immunoprecipitation; WT, wild‐type; **P < 0.01, ***P < 0.001

Long non‐coding RNAs can regulate miRNA activity by acting as either competitive endogenous RNAs or miRNA sponges.^17^ Generally, miRNAs function as ribonucleoprotein particles (RNPs), which are subsequently rearranged into RNA‐induced silencing complexes (RISC). As reported, each RISC contains a member of the Argonaute (Ago) protein family that includes Ago1‐4, which acts as a catalytic component. It has been demonstrated that in human the system, only the Ago2 protein‐containing RNPs show RISC activity.^18^ RIP assays showed that H19 was more enriched to the Ago2 protein when compared to IgG protein (*P < *0.001, Figure 2A). These results suggest the “sponge” function of lncRNA H19.

As previously mentioned, H19 and miRNA‐106b‐5p binding were hypothesized using various predictive tools (Figure 2B). Our RNA pull‐down assays supported these predictions, as the results revealed that H19 would bind to the predicted binding region of homeostatic miRNA‐106b‐5p, but not when the region was artificially mutated (Figure 2B,C, *P < *0.001). The direct interaction of H19 and miRNA‐106‐5p was further verified by luciferase assays (Figure 2D). We constructed two H19‐related luciferase reporters, one with the wild‐type (WT) predicted binding region for miRNA‐106b‐5p, while the other with the mutated sequences (MT; Figure 2B). Each construct was co‐transfected into 293T cells with miRNA‐106b‐5p mimics. We found that miRNA‐106b‐5p mimics dramatically suppressed the luciferase activity of H19‐WT reporter by approximately 74% (*P < *0.001), while the inhibitory rate for H19‐MT was merely 3% (Figure 2D).

Finally, to test the cellular effect of H19 on miRNA‐106b‐5p, we knocked down H19 in TCam‐2 cells. Compared to the control TCam‐2 cells, qPCR results showed that miRNA‐106b‐5p was significantly upregulated in the H19‐knockdown cells (Figure 2E, *P < *0.01). Collectively, these results indicated that H19 might function as an endogenous miRNA‐106b‐5p sponge in seminoma cells.

### 3.3 The effect of H19 and miRNA‐106b‐5p interactions on TDRG1

**Figure 3:**
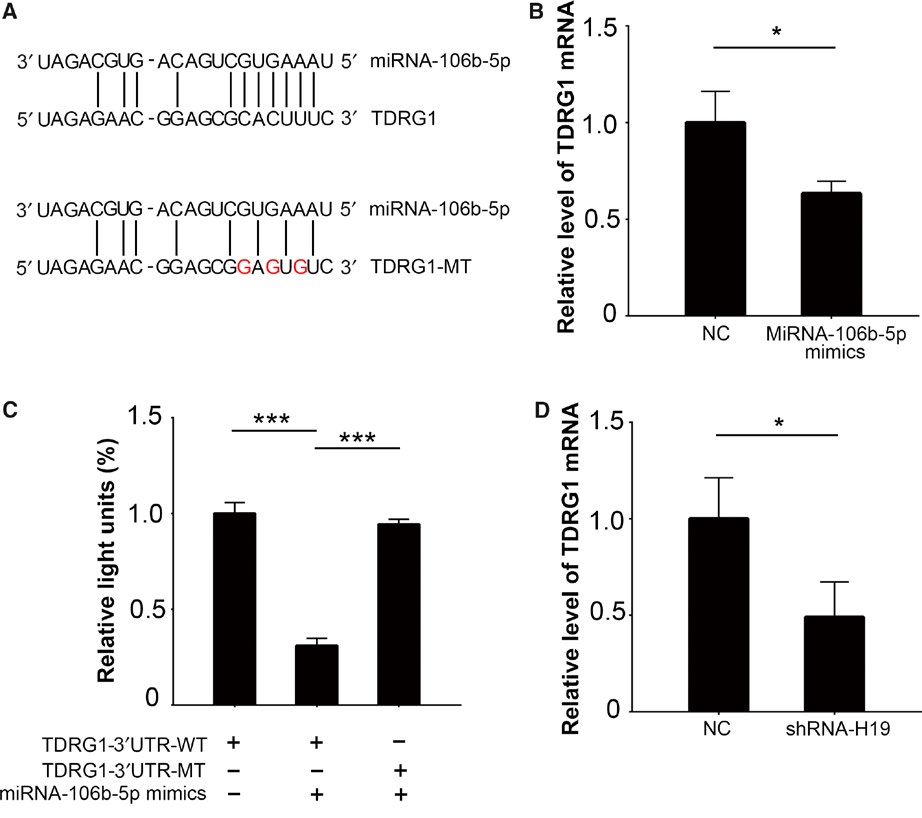
MiRNA‐106b‐5p attenuates the expression of TDRG1. A, A schematic diagram of the predicted binding sites of miRNA‐106b‐5p with TDRG1. The artificially mutated sequences in TDRG1 are also shown. B, MiRNA‐106b‐5p was overexpressed and the level of TDRG1 mRNA was detected using qPCR. C, 3′UTR‐TDRG1‐WT reporters or 3′UTR‐TDRG1‐MT reporters were co‐transfected into 293T cells with miRNA‐106b‐5p mimics and Renilla plasmids, and then luciferase activities were measured. Firefly luciferase activity was normalized to Renilla. D, H19 was knocked down and the level of TDRG1 mRNA was measured by qPCR. 3′UTR, 3′‐untranslated regions; *P < 0.05, ***P < 0.001

As previously stated, TDRG1 was predicted to be one of the targets of miRNA‐106b‐5p (Figure 3A). To investigate the effect of miRNA‐106b‐5p on TDRG1 expression, we transfected miRNA‐106b‐5p mimics into TCam‐2 cells. Compared to the control group, TDRG1 was significantly downregulated in miRNA‐106b‐5p overexpressed TCam‐2 cells (Figure 3B, *P < *0.05). We then mutated the miRNA‐106b‐5p binding sites in TDRG1‐3′UTR (Figure 3A) and performed luciferase assays to verify their interactions. miRNA‐106b‐5p expression was significantly suppressed the luciferase activity of TDRG1‐WT compared to TDRG1‐MT (*P < *0.001; Figure 3C). As H19 targets miRNA‐106b‐5p, we assumed that H19 might affect the expression of TDRG1. Based on qPCR results, TDRG1 mRNA levels were found to be downregulated in the H19‐knockdown TCam‐2 cells compared to control cells (Figure 3D, *P < *0.05). Combining the previous results, we demonstrated that H19 promoted the expression of TDRG1 by sequestering miRNA‐106b‐5p in seminoma cells.

### 3.4 MiRNA‐106b‐5p reintroduces sensitivity of TCam‐2/CDDP cells to CDDP treatment

**Figure 4:**
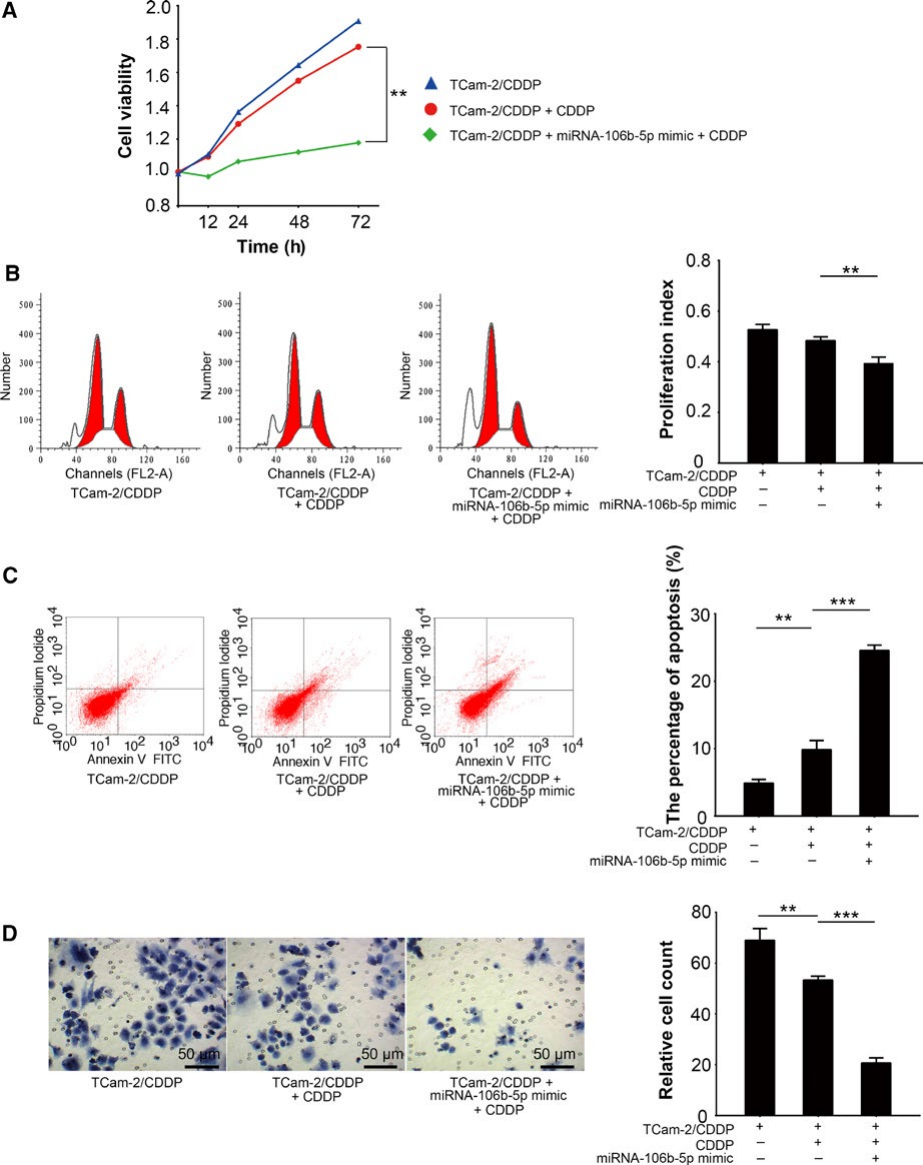
MiRNA‐106b‐5p promotes cellular sensitivity to CDDP in TCam‐2/CDDP cells. MiRNA‐106b‐5p was overexpressed in TCam‐2/CDDP cells and cellular responses to CDDP treatment (3 µmol/L) were investigated. A, Cell viability was measured by MTT assay 0, 12, 24, 48, and 72 h after treatment. Seventy‐two hours after drug treatment, both distribution of cell cycle (proportion of sub G0, G0/G1, S, and G2/M phases) (B) and quantity of apoptotic cells (C) were analyzed by FCM assays. D, Cellular invasiveness was determined by Matrigel transwell assays. The cells penetrated the Matrigel were fixed and counted 72 h after drug treatment. FCM, flow cytometry; MTT, 3‐(4,5‐dimethylthiazol‐2‐yl)‐2,5‐diphenyltetrazolium bromide; **P < 0.01, ***P < 0.001

Because TDRG1 can modulate seminoma cell sensitivity to CDDP, as previously reported,^6^ we further assessed the impact of miRNA‐106b‐5p on the response of TCam‐2 cells to CDDP. We transfected miRNA‐106b‐5p mimics into TCam‐2/CDDP cells and performed MTT assays to test cell viability in a CDDP environment (Figure 4A). As expected, miRNA‐106b‐5p mimics attenuated the viability of TCam‐2/CDDP cells after 72 hours (*P < *0.01; Figure 4A), which indicated that miRNA‐106b‐5p reintroduced sensitivity of TCam‐2/CDDP cells to CDDP treatment. Considering the fact that both cell cycle and cell apoptosis may contribute to altered cellular viability, we also measured the distribution of the cell cycle and the percentage of apoptotic cells by performing FCM assays using the same treatment condition as mentioned above. We observed that miRNA‐106b‐5p mimics enhanced the efficacy of CDDP on TCam‐2/CDDP cells, which was reflected in a further decrease in PI (Figure 4B, *P < *0.01), and a more than twofold increase in apoptotic cells induction (Figure 4C, *P < *0.001). Furthermore, miRNA‐106b‐5p also significantly reduced the number of TCam‐2/CDDP cells penetrating the Matrigel in cell invasion assays under CDDP treatment (Figure 4D, *P < *0.001). Together, based on this evidence, miRNA‐106b‐5p elevates the sensitivity of CDDP‐resistant seminoma cells to CDDP, which may be due to the suppression of TRGD1 expression by miRNA‐106b‐5p in seminoma cells.

### 3.5 Knockdown of H19 promotes the sensitivity of TCam‐2/CDDP cells

**Figure 5:**
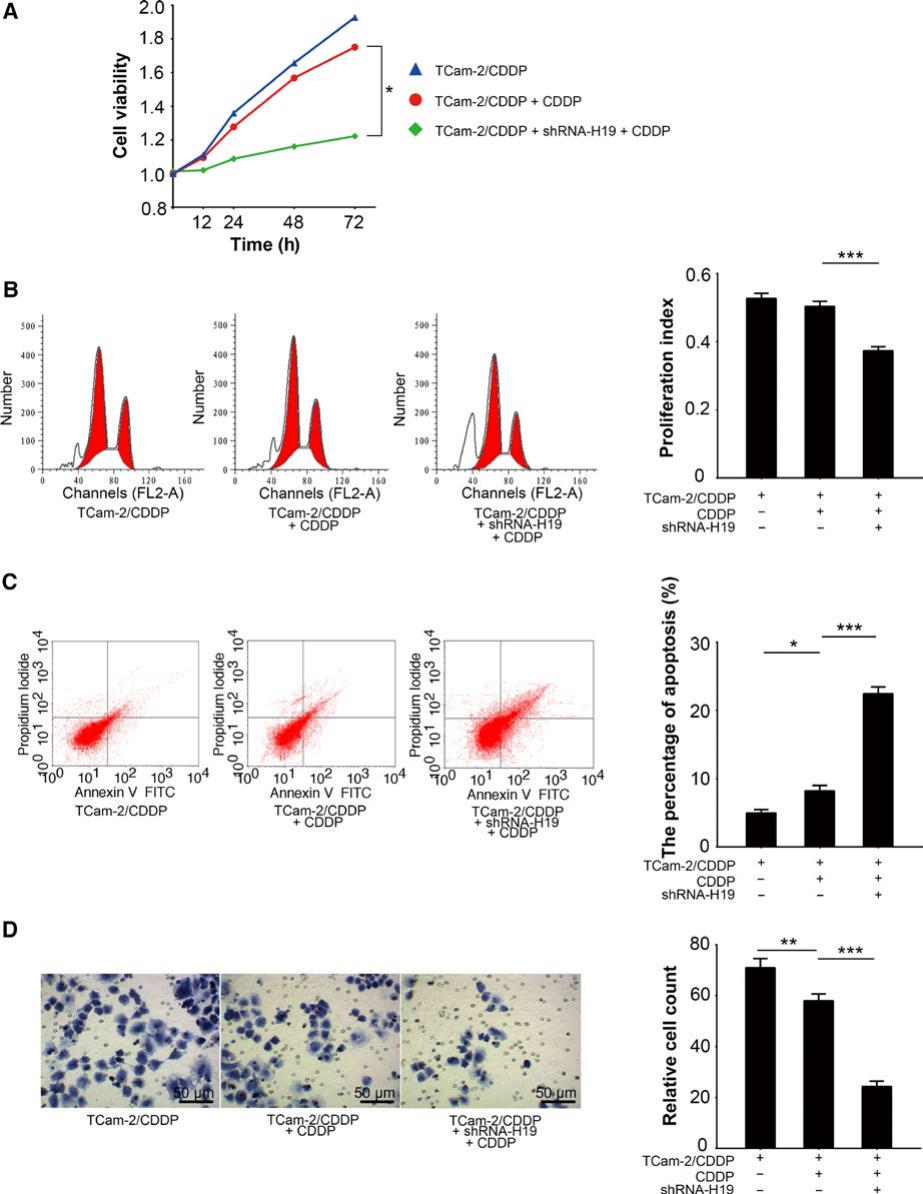
H19 attenuates cellular sensitivity to cisplatin in TCam‐2/CDDP cells. H19 was silenced in TCam‐2/CDDP cells and the impact on cells was studied in CDDP treatment (3 µmol/L) context. A, Cell viability was measured by MTT assay 0, 12, 24, 48 and 72 h after treatment. Seventy‐two hours after drug treatment, both distribution of cell cycle (proportion of sub G0, G0/G1, S, and G2/M phases) (B) and quantity of apoptotic cells (C) were analyzed by FCM assays. D, Cellular invasiveness was determined by Matrigel transwell assays. The cells penetrated the Matrigel were fixed and counted 72 h after drug treatment. *P < 0.05, **P < 0.01, ***P < 0.001

The “sponge” effect of H19 on miRNA‐106b‐5p has already been confirmed in TCam‐2 cells as described above. Based on these findings, we hypothesized that H19 might have an opposite function of miRNA‐106b‐5p on TCam‐2/CDDP cells. In fact, TCam‐2/CDDP cells with decreased H19 expression showed a higher response to CDDP treatment than controlled TCam‐2/CDDP cells using MTT assays (Figure 5A). Additionally, in contrast to miRNA‐106b‐5p, H19 attenuated the antitumor effect of CDDP in TCam‐2/CDDP cells. Results from cell cycle assays and cell apoptosis assays demonstrated that knockdown of H19 in TCam‐2/CDDP cells inhibited the cell cycle progression (Figure 5B, *P < *0.001) and enhanced apoptosis (Figure 5C, *P < *0.001) in drug treatment condition. Moreover, knockdown of H19 also mimicked the effect of miRNA‐106b‐5p in cell invasiveness (Figure 5D, *P < *0.001). Together, these results support the idea that H19 provides seminoma cells with an ability to survive under CDDP treatment, which may be determined by the “sponge” effect to sequester miRNA‐106b‐5p.

### 3.6 In vivo effect of the H19/miRNA‐106b‐5p/TDRG1 axis on CDDP sensitivity in seminoma

Seminoma cells were subcutaneously injected into mice with or without CDDP treatment. Consistent with the results from the in vitro experiments, in vivo TCam‐2/CDDP cells under CDDP treatment grew more rapidly than TCam‐2 cells under CDDP treatment (Figure S5A). This was also confirmed by H&E staining of the tumor tissues and the IHC staining of proliferation marker Ki‐67 in the xenografts (Figure S5B,C). Together, these data indicated that TCam‐2/CDDP cells are also resistant to CDDP treatment in an in vivo environment.

**Figure 6:**
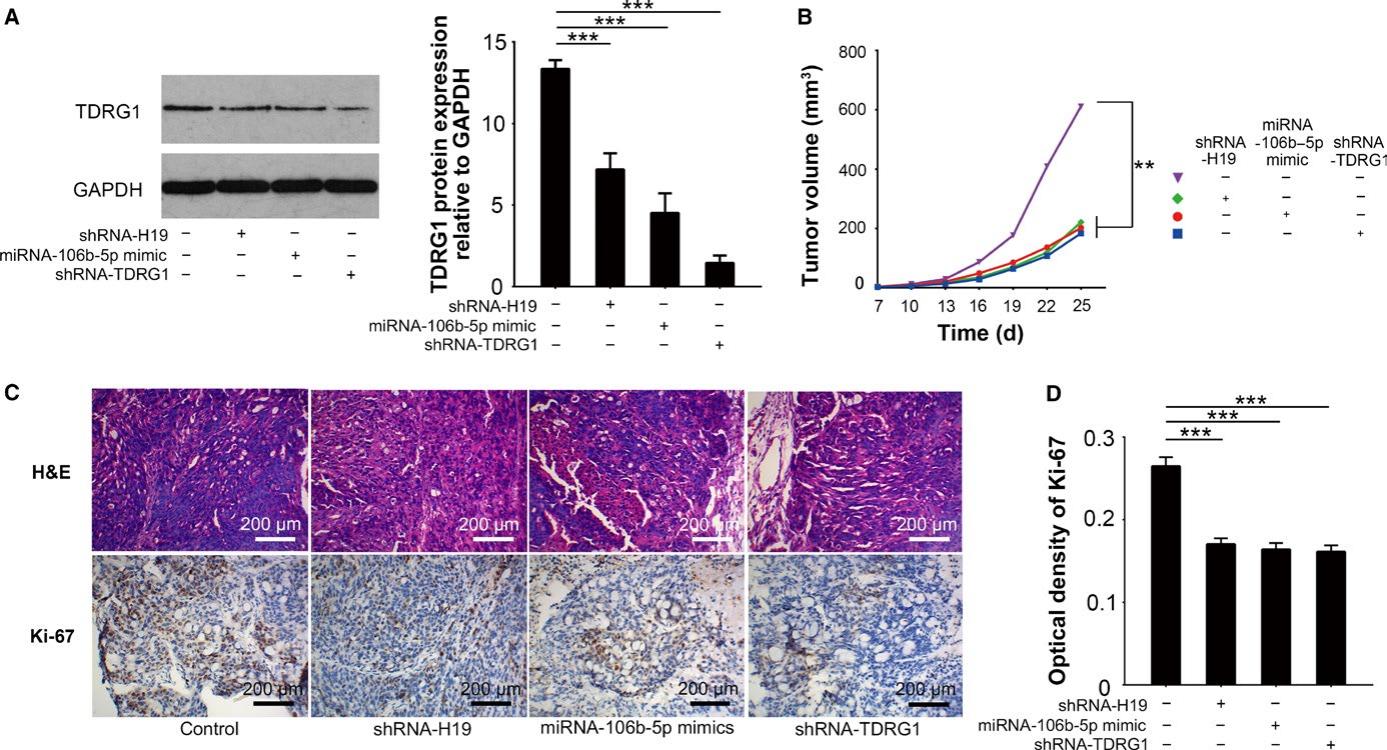
The effect of H19/miRNA‐106b‐5p/TDRG1 axis on CDDP sensitivity of seminoma in vivo. Male mice were subcutaneously injected with tumor cells to establish xenografts and then intraperitoneally injected with CDDP (6 mg/kg body weight) or vehicle every week for three doses. The mice were humanely sacrificed and their tumors were excised 25 d after inoculation. A, The in vivo effects of H19 and miRNA‐106b‐5p on TDRG1 expression were analyzed by immunoblotting. GAPDH served as a loading control. B, Tumor volumes were measured every third day for consecutive seven times. C, Representative photographs of H&E staining of tumor tissues and IHC staining of Ki‐67 are presented. D, The effects of H19, miRNA‐106b‐5p, and TDRG1 on Ki‐67 expression of TCam‐2/CDDP cells were analyzed by IHC assays. H&E, hematoxylin and eosin; IHC, immunohistochemistry; **P < 0.01, ***P < 0.001

The in vivo effect of the H19/miRNA‐106b‐5p/TDRG1 axis on CDDP sensitivity was also explored in TCam‐2/CDDP cells. We found that not only the knockdown of H19 (Figure S6A), but also the artificially introduction of miRNA‐106b‐5p mimics (Figure S6B) significantly decreased the expression of TDRG1 protein in xenografts derived from TCam‐2/CDDP cells (Figure 6A). Knockdown of H19 also resulted in higher level of miRNA‐106b‐5p in vivo (Figure S6B, *P < *0.05). More interestingly, in favor of the direct knockdown of TDRG1 expression, both knockdown of H19 and introduction of miRNA‐106b‐5p mimics recovered the sensitivity of TCam‐2/CDDP cells to CDDP in vivo (Figure 6B‐D). Collectively, we demonstrated the existence of the H19/miRNA‐106b‐5p/TDRG1 axis and uncovered their contribution to in vivo CDDP resistance.

## 4 DISCUSSION

**Figure 7:**
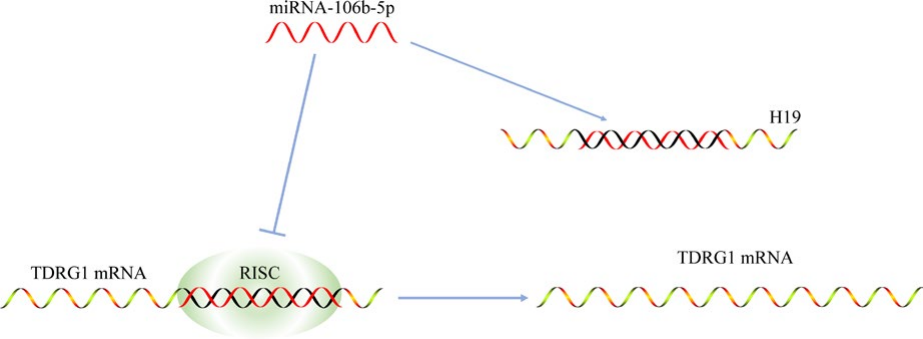
A schematic diagram of H19 acting as a sponge of miRNA‐106b‐5p and rescuing the expression of TDRG1. In the absence of H19, mature miRNA‐106b‐5p silences the function of TDRG1 through loading in RISC. After the introduction of H19, high level of H19 sequesters miRNA‐106b‐5p and rescues the expression of TDRG1. RISC, RNA‐induced silencing complex

Seminoma represents a successful paradigm of curative cancer.^19^ As seminoma cells are generally highly sensitive to platinum drugs‐based therapies, the majority of patients, even those with metastatic diseases, usually have a positive prognosis or are cured after treatment. However, a few CDDP‐resistant cases have also reported, usually with a poor outcome.^20^ Several mechanisms underlying the high sensitivity or the resistance of seminoma cells to CDDP have been reported.^21^, ^22^ One of these mechanisms reported involved the TDRG1 gene, which can modulate the activity of PI3K/Akt signaling pathway to regulate CDDP sensitivity.^6^ In this study, we expanded our knowledge of TDRG1 in seminoma and cellular responses to CDDP. The H19/miRNA‐106b‐5p/TDRG1 axis was validated in homeostatic seminoma as well as in a CDDP‐resistant context. H19 was found to act as a “sponge” to decrease the amount of TDRG1 mRNA exposed to miRNA‐106b‐5p (Figure 7). Whether interrupting this axis will provide new targets to treat seminoma, or even cisplatin‐resistant diseases, warrants further investigation.

H19, previously known as an imprinted oncofetal gene, was first identified in embryonic development processes, in which the expression of H19 was downregulated postnatally.^23^ Studies on H19 originally focused in the field of genomic imprinting, while current research is now investigating its biological function in coding a type of ncRNA, lncRNA H19. Despite much controversies on its roles in tumorgenesis,^23^, ^24^ accumulating data have highlighted the importance of H19 in tumor initiation and progression.^25^, ^26^, ^27^ Furthermore, research on the role of H19 as a cellular self‐protective response to stress conditions, such as chemotherapy, is emerging.^23^ It was reported that H19 expression is established in CDDP‐resistant serous ovarian cancer and contributes to CDDP resistance, which indicates that changes in the expression profiles of lncRNAs, including overt H19 expression, may be a strategy of tumor cells to survive chemotherapy.^15^ Our findings that H19 renders seminoma cells with the CDDP‐resistant ability support this theory. More interestingly, seminoma stands at the crossroads of developmental and neoplastic processes,^28^ which, at least partially, explains our findings that H19 is overexpressed in seminoma. However, more evidence is still needed to uncover the tumorigenic property of H19 in seminoma.

The role of miRNA‐106b‐5p in tumorigenesis remains controversial. Overt miRNA‐106b‐5p was validated in several types of malignant disease such as gastric,^29^ colorectal,^30^ and hepatocellular cancer.^31^ In these studies, miRNA‐106b‐5p was reported to promote tumor cell proliferation, indicating its carcinogenic role in these cancers. However, there are some opposing views in the literature. Ni et al^32^ reported that miRNA‐106b‐5p is significantly repressed in metastatic sites, such as in bones, compared to the original breast tumor. Consistently, a study from Dong et al^33^ demonstrated that miRNA‐106b‐5p is dramatically deceased in endometrial tumor cells with highly invasive properties. These studies reveal to us that miRNA‐106b‐5p may also play a role as a tumor suppressor. Here, the findings in seminoma support the latter, as we found that miRNA‐106b‐5p decreases the expression of TDRG1 at the post‐transcriptional level. At least partially, the different roles of miRNA‐106b‐5p can be explained by different cellular contexts, while more detailed mechanisms need to be further studied.

An increasing amount of studies have found evidence to support the idea that there are interactions between lncRNAs and miRNAs. The findings from this paper may uncover a novel potential target for cancer therapy. lncRNAs acting as a “sponge” to attract certain miRNAs exemplifies a form of these interactions.^17^, ^34^ In this type of “sponge” effect, lncRNAs can sequester their targeted miRNAs and inhibited their functions.^35^ The role of H19 as a miRNA sponge was previously reported in epithelial‐mesenchymal transitions in colorectal cancer.^36^ In the present study, we found that H19 was highly connected with the important component in miRNAs‐related RISCs, which highly implies the “sponge” function of H19 and that H19 is presumably located in cytoplasm in seminoma. However, whether H19 can attract miRNAs other than miRNA‐106b‐5p in seminoma is still unclear.

It should be noted that, despite the aforementioned original findings, some limitations are also inevitable. Due to the difficult nature of collecting tumor tissues from CDDP‐resistant seminoma patients, it was challenging to directly compare the levels of H19, miRNA‐106b‐5p, and TDRG1 between primary tumors and their CDDP‐resistant counterparts. However, the significant associations between TDRG1 and the two ncRNAs in seminoma, combined with their differential expression between normal and tumor tissues as well as between CDDP‐resistant tumor cells and their origins, may help us to understand their roles in CDDP sensitivity. Furthermore, though the TCam‐2 cell line has already been confirmed to be a valid model for seminoma,^37^, ^38^ only this one cell line was used in this study due to difficulty in primary culture as well as difficulty in accessing other noncommercial seminoma cell lines. Further research using more seminoma cell lines is needed.

In summary, our studies demonstrated that elevated H19 and decreased miRNA‐106b‐5p expression are correlated with CDDP resistance in seminoma cells. The “sponge” effect of H19 promotes the expression of TDRG1 through sequestering miRNA‐106b‐5p. Furthermore, the newly identified H19/miRNA‐106b‐5p/TDRG1 axis may serve as a potential target for the treatment of seminoma and the CDDP‐resistant tumors.

## CONFLICT OF INTEREST

None declared.

## DATA ACCESSIBILITY

The data generated and analyzed during this study are available upon reasonable request from the corresponding author.
