# Supplementary figures and images for: Long non‐coding RNA H19 promotes TDRG1 expression and cisplatin resistance by sequestering miRNA‐106b‐5p in seminoma
Source: Cancer Med. 2018 Nov 14;7(12):6247–57. doi: 10.1002/cam4.1871 (PMC6308085; doi:10.1002/cam4.1871)

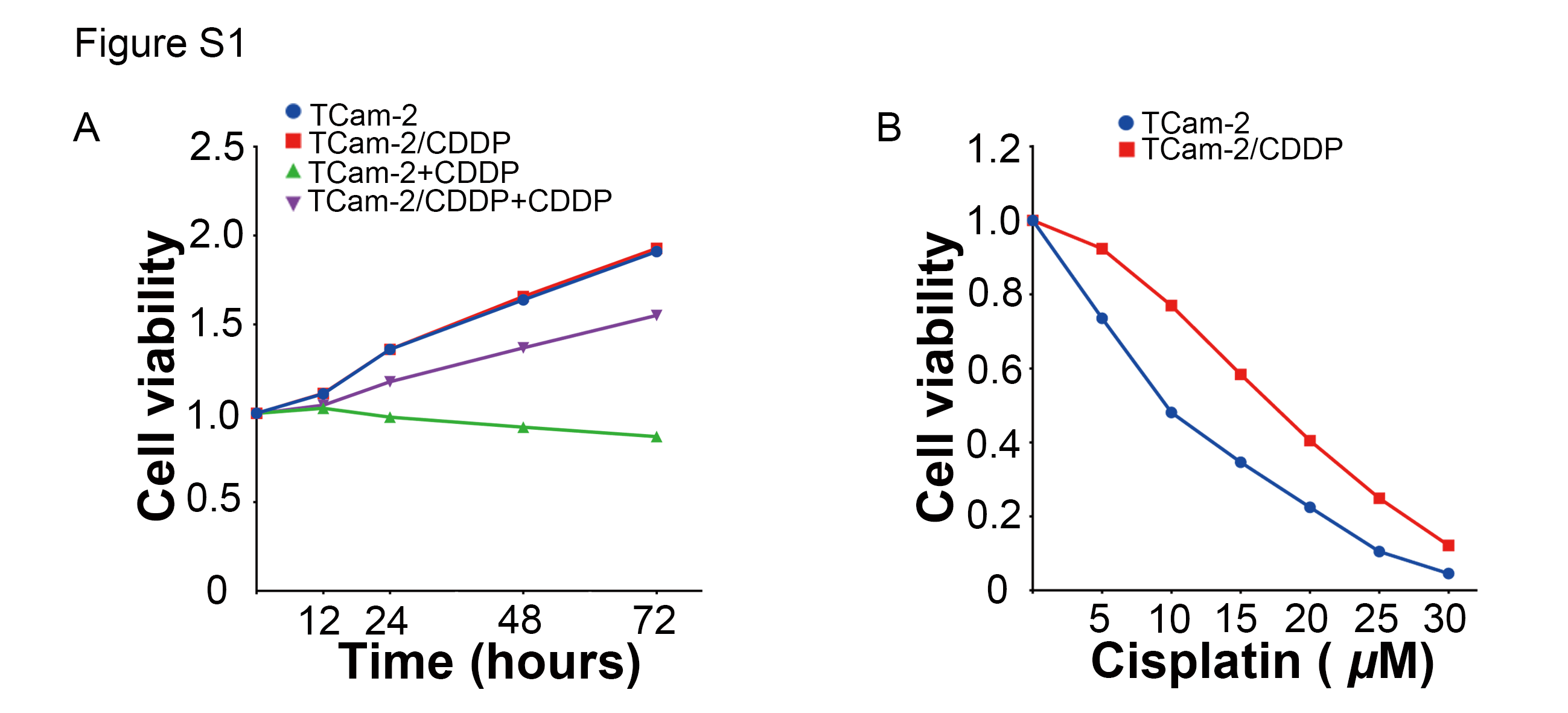

Supplement: Supplementary file 1 [file CAM4-7-6247-s001.tif]

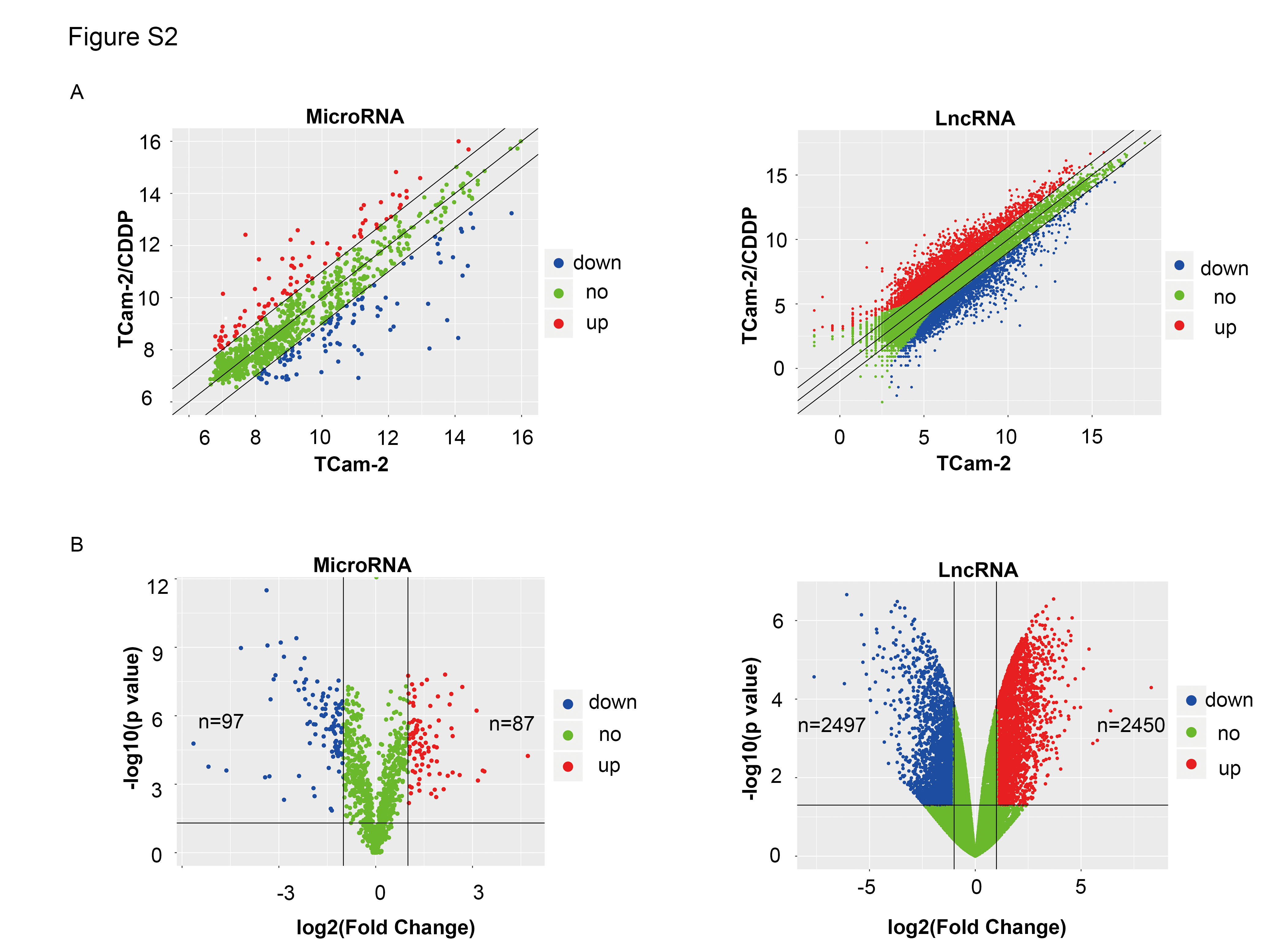

Supplement: Supplementary file 2 [file CAM4-7-6247-s002.tif]

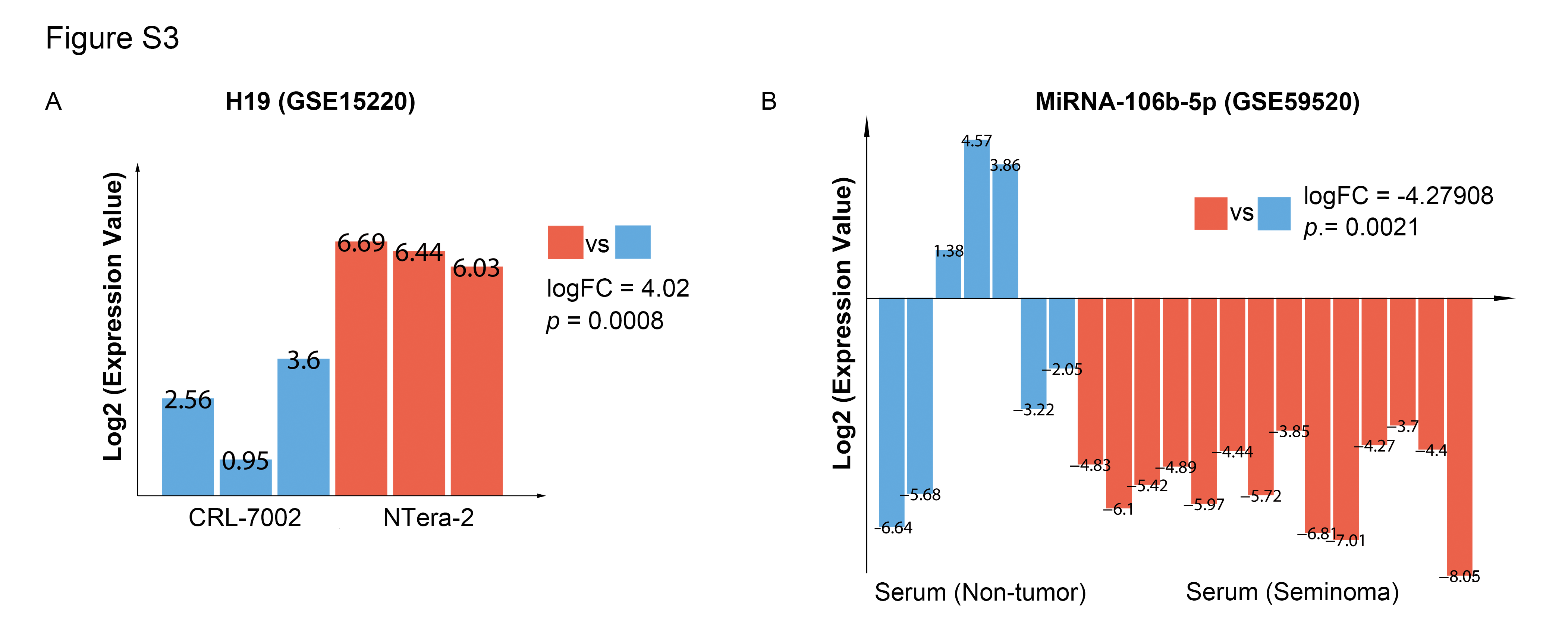

Supplement: Supplementary file 3 [file CAM4-7-6247-s003.tif]

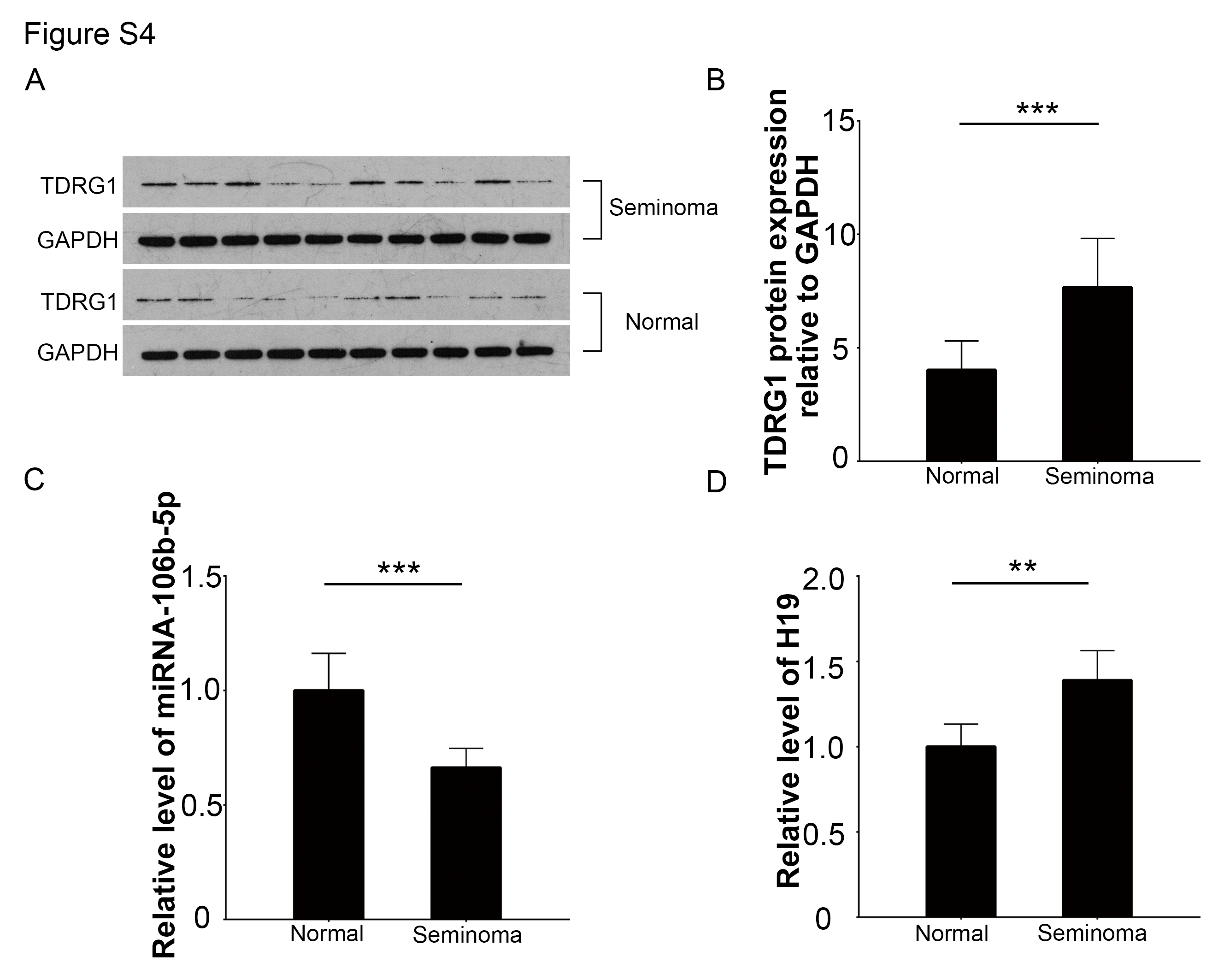

Supplement: Supplementary file 4 [file CAM4-7-6247-s004.tif]

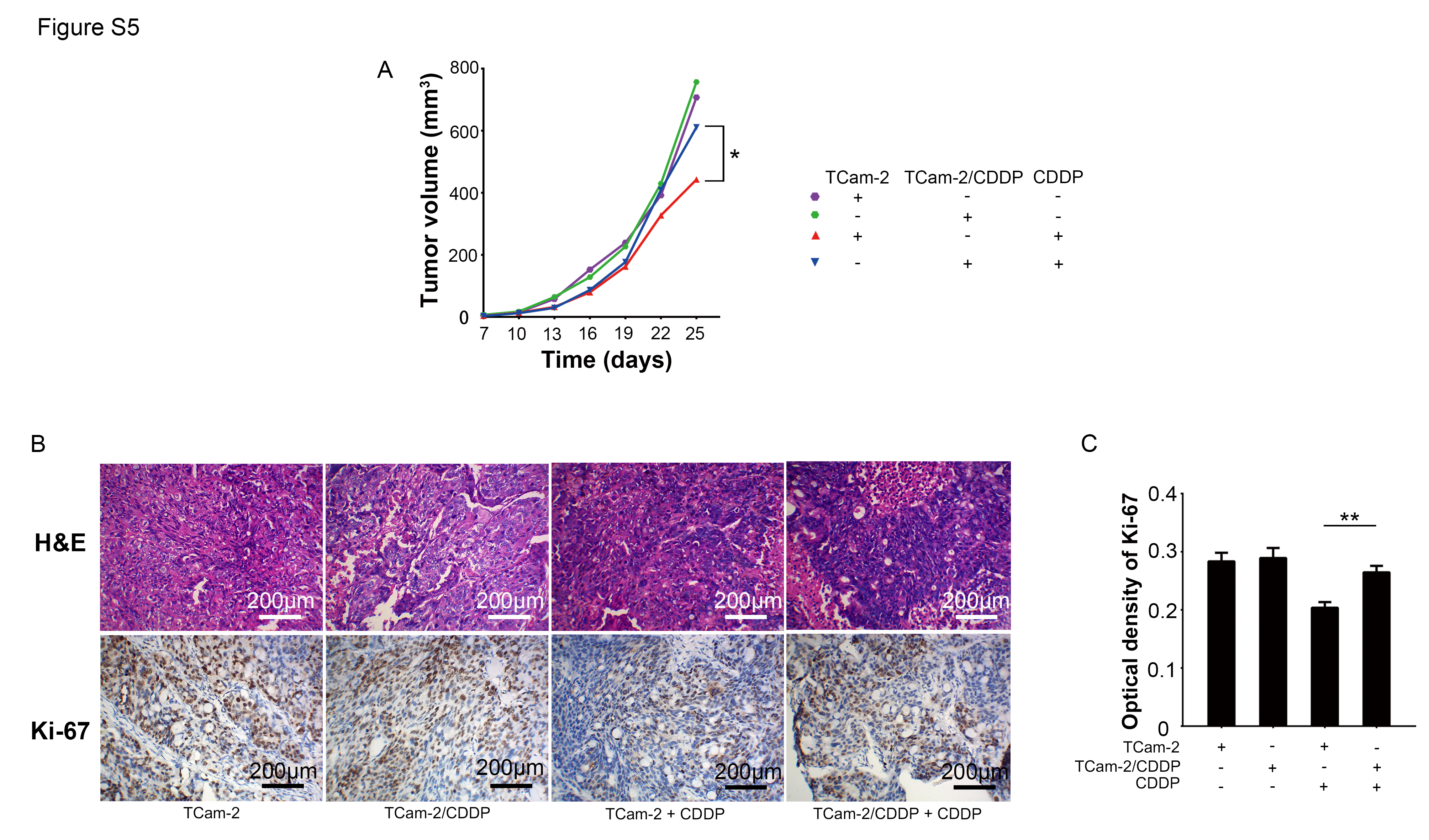

Supplement: Supplementary file 5 [file CAM4-7-6247-s005.tif]

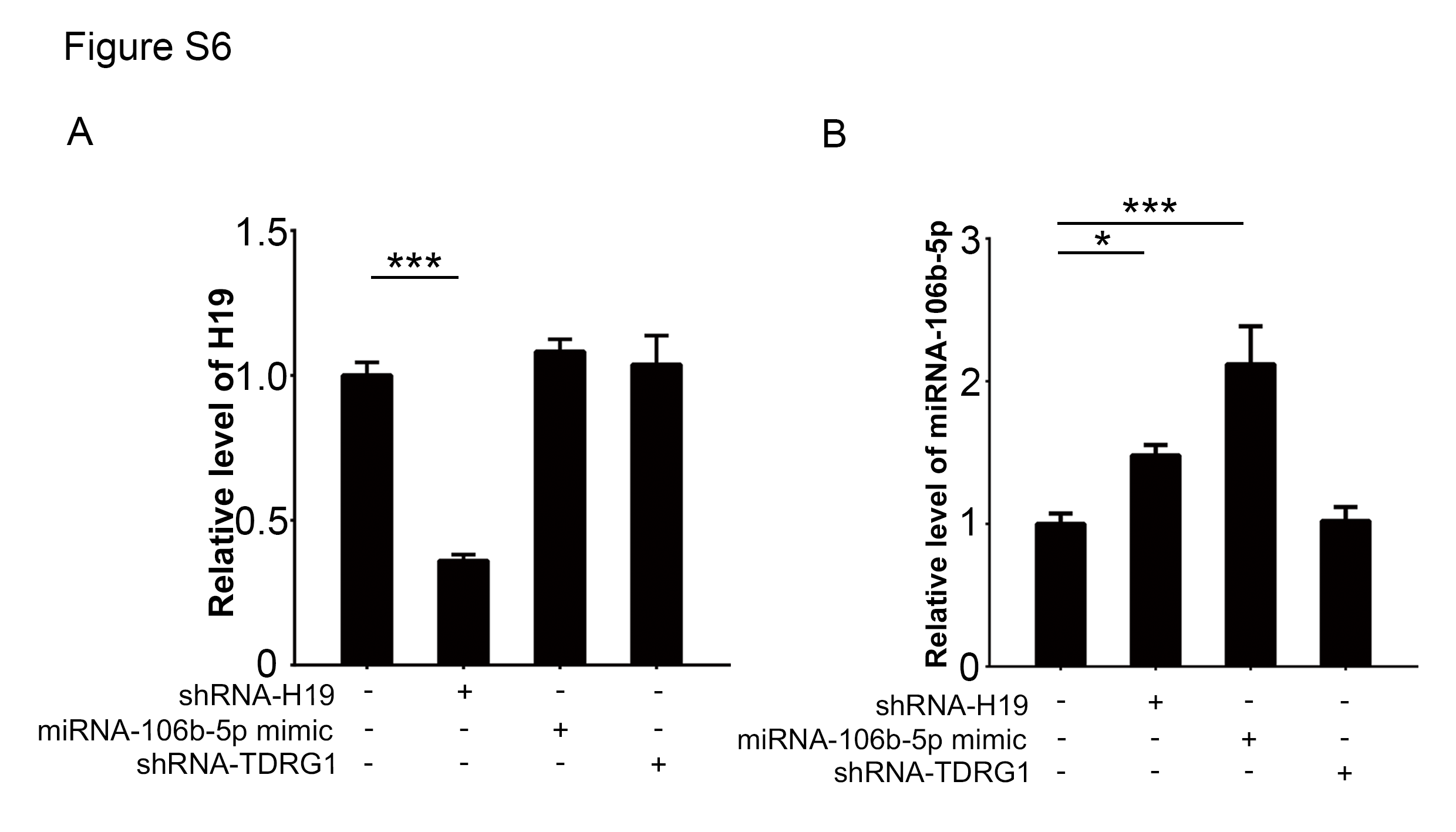

Supplement: Supplementary file 6 [file CAM4-7-6247-s006.tif]
