# Supplementary material for: Long non‐coding RNA H19 promotes TDRG1 expression and cisplatin resistance by sequestering miRNA‐106b‐5p in seminoma
Source: Cancer Med. 2018 Nov 14;7(12):6247–57. doi: 10.1002/cam4.1871 (PMC6308085; doi:10.1002/cam4.1871)
Supplement: Supplementary file 7 [file CAM4-7-6247-s007.docx]

Table S1. The list of primers and reference sequences

| Name | Sequence (5’ to 3’) |
| --- | --- |
| primer for miRNA-106b-5p F | ACACTCCAGCTGGGTAAAGTGCTGACAGT |
| primer for miRNA-106b-5p R | CTCAACTGGTGTCGTGGAGTCGGCAATTCAGTTGAGATCTGCAC |
| primer for H19 F | TGCTGCACTTTACAACCACTG |
| primer for H19 R  primer for TDRG1 F  primer for TDRG1 R | ATGGTGTCTTTGATGTTGGGC  GAAGAGGAGGGAGGCAGTCT  GGGAACCTAGACCTGGGAAG |
| primer for U6 F | CTCGCTTCGGCAGCACA |
| primer for U6 R | AACGCTTCACGAATTTGCGT |
| primer for GAPDH F | GGAGCGAGATCCCTCCAAAA |
| primer for GAPDH R | GGCTGTTGTCATACTTCTCATGG |
| shRNA-H19 |  |
| target sequence | CCAACATCAAAGACACCAT |
| template F | CACCGCCAACATCAAAGACACCATTTCAAGAGAATGGTGTCTTTGATGTTGGTTTTTTG |
| template R | GATCCAAAAAACCAACATCAAAGACACCATTCTCTTGAAATGGTGTCTTTGATGTTGGC |
| scrambled control F | CACCGTTCTCCGAACGTGTCACGTCAAGAGATTACGTGACACGTTCGGAGAATTTTTTG |
| scrambled control R | GATCCAAAAAATTCTCCGAACGTGTCACGTAATCTCTTGACGTGACACGTTCGGAGAAC |
| miRNA-106b-5p mimic |  |
| miRNA-106b-5p-wild type | UAAAGUGCUGACAGUGCAGAU |
| miR-106b-5p-mutant type | UGACUUCCUGACAGUGCAGAU |
| scrambled control | UUGUACUACACAAAAGUACUG |
