# Supplementary material for: Long non‐coding RNA H19 promotes TDRG1 expression and cisplatin resistance by sequestering miRNA‐106b‐5p in seminoma
Source: Cancer Med. 2018 Nov 14;7(12):6247–57. doi: 10.1002/cam4.1871 (PMC6308085; doi:10.1002/cam4.1871)
Supplement: Supplementary file 9 [file CAM4-7-6247-s009.doc]

**RECOMBINANT PLASMID CONSTRUCT REPORT**

**Project Name**: MUTANT 3'UTR of TDRG1

**Insert Sequence Informatoin**:

| Gene/insert name: | MUTANT 3'UTR of TDRG1 |
| --- | --- |
| Official Symbol: | TDRG1 |
| Gene ID: | 732253 |
| RefSeq: | NR_024015 |
| Organism: | Homo sapiens |
| 5'Primers: | aagatcaggactgctgaaagaat |
| 3'Primers: | acggaaagagtcaggcctggg |
| Insert size: | 474 nt |
| Insert sequence: | aagatcaggactgctgaaagaatgaagaagaacttacttggcctaggatgtggctagagaacggagcGgAgTgTcacttacctggcaaggtggttttggaagtttccacggaagccttcccagggccgctgctgcccgtcctcgcctaggtgtggagcttcccgaccggctggggagggaatgtcctgcgggagccgccgaggaccctctttagcctcttccctggcttggctgcagctgcagtctggtactcgcccattctgctcttcttcacctccgtattttctctctcacggaagaagaattccattctcccatcccaggaaggagagtgccctgcgtgccacgaaagagcccaggacccacaggacaaatacgtcactggcagacctgccttcgccagcgccagcccatctctgaaacctccagcatttgtgccccggtccggcccctcccaggcctgactctttccgt |

**Vector Information**:

| Vector backbone: | pmirGLO |
| --- | --- |
| Backbone manufacturer: | Promega |
| Vector type: | Mammalian Expression, Dual-Luciferase miRNA Target Expression Vector |
| Bacterial resistance: | Ampicillin |
| Selectable markers: | neo |
| Cloning site | 5' XhoI –3' SalI |

**Validation Information**:

| Validated by PCR |  |
| --- | --- |
| Validated by Sequencing |  |

**Plasmid Prepration Information**:

Growth strain: DH5α [*fhuA2 Δ(argF-lacZ)U169 phoA glnV44 Φ80 Δ(lacZ)M15 gyrA96 recA1 relA1 endA1 thi-1 hsdR17*]

Growth medium: LB

Growth antibiotics: Ampicillin

Growth temperature: 37C

DNA prepration by: QIAGEN Plasmid Mini Kit (*Cat#12125*)
